# Gut microbiota and Parkinson’s disease: potential links and the role of fecal microbiota transplantation

**DOI:** 10.3389/fnagi.2024.1479343

**Published:** 2024-11-29

**Authors:** Maosen Feng, Zhiyan Zou, Pingping Shou, Wei Peng, Mingxue Liu, Xiaoan Li

**Affiliations:** ^1^NHC Key Laboratory of Nuclear Technology Medical Transformation, Mianyang Central Hospital, School of Medicine, University of Electronic Science and Technology of China, Mianyang, China; ^2^Department of Gastroenterology, National Clinical Key Specialty, Mianyang Central Hospital, School of Medicine, University of Electronic Science and Technology of China, Mianyang, China; ^3^School of Life Sciences and Engineering, Southwest University of Science and Technology, Mianyang, China

**Keywords:** fecal microbiota transplantation, gut-brain axis, gut microbiota, Parkinson’s disease, α-synuclein

## Abstract

Parkinson’s disease (PD) is the second most common neurodegenerative disease worldwide and seriously affects the quality of life of elderly patients. PD is characterized by the loss of dopaminergic neurons in the substantia nigra as well as abnormal accumulation of α-synuclein in neurons. Recent research has deepened our understanding of the gut microbiota, revealing that it participates in the pathological process of PD through the gut-brain axis, suggesting that the gut may be the source of PD. Therefore, studying the relationship between gut microbiota and PD is crucial for improving our understanding of the disease’s prevention, diagnosis, and treatment. In this review, we first describe the bidirectional regulation of the gut-brain axis by the gut microbiota and the mechanisms underlying the involvement of gut microbiota and their metabolites in PD. We then summarize the different species of gut microbiota found in patients with PD and their correlations with clinical symptoms. Finally, we review the most comprehensive animal and human studies on treating PD through fecal microbiota transplantation (FMT), discussing the challenges and considerations associated with this treatment approach.

## 1 Introduction

Parkinson’s disease (PD) is a degenerative neurological disorder commonly affecting the elderly. In its later stages, PD typically presents with tremors, muscle rigidity, akinesia, and postural instability (Bloem et al., 2021). The core pathological features of PD include the abnormal aggregation of α-synuclein and the degeneration and necrosis of dopaminergic neurons in the substantia nigra (Hansson, 2021). The incidence of PD is primarily associated with age, environment, lifestyle, and genetic factors, with genetic and environmental factors playing significant roles. However, no specific etiology has been definitively linked to PD (Tansey et al., 2022). The pathogenesis of PD is complex, involving mechanisms such as abnormal aggregation of α-synuclein, mitochondrial dysfunction, lysosomal or vesicular transport issues, synaptic transmission disorders, and neuroinflammation (Kalia and Lang, 2015).

In recent years, most studies have found that gut microbiota have closely related to the occurrence of human diseases. The gut microbiota are not static, and they are dynamic changes due to the genetic background, diet, lifestyle, drug use, age, and environmental factors of the host (Fan and Pedersen, 2021). Therefore, under the above factors, the dynamic changes of the gut microbiota may participate in the pathogenesis of the PD through the gut-brain axis. As early as 2006, it was suggested that, under certain conditions, several bacteria could multiply and aggregate α-synuclein in the gastric mucosa, which then reaches the central nervous system through complex conduction pathways, contributing to the pathogenesis of PD. This hypothesis proposed that PD may originate in the gut (Braak et al., 2006). Subsequently, Holmqvist et al. (2014) demonstrated that α-synuclein could be transmitted from the gut to the brain through the vagus nerve, causing PD motor symptoms. These studies underscore the importance of the gut-brain axis in PD progression.

Previously, it was believed that inflammatory mediators and neurohormones mediate the brain and gut communicate bidirectionally (Agirman et al., 2021). Recent studies, however, have shown a close relationship between gut microbiota and the development of PD. The gut microbiota and its metabolites participate in the interaction between the gut-brain axis of PD, forming what is now known as the microbiome-gut-brain axis (Agirman and Hsiao, 2021). Moreover, there is a significant difference in gut microbiota between people with PD and healthy people, with correlations observed between key microbiota categories and PD symptoms (Heinzel et al., 2020; Palacios et al., 2023).

At present, the primary treatment for PD involves dopamine replacement therapy to alleviate motor symptoms and has no effect on non-motor symptoms. Recent research indicates that levodopa can reduce the Aβ pathology in the Alzheimer mice model (Lee et al., 2024). But, there is no evidence that Levodopa can reduce the deposition of α-Synuclein in PD. Given the potential link between gut microbiota and PD, many new therapies targeting the regulation of gut microbiota have emerged. A number of clinical diseases have been treated with fecal microbiota transplantation (FMT) and certain therapeutic effects have been observed (Smits et al., 2013). FMT can help restore the normal composition of gut microbiota, mediate the bidirectional interaction of the gut-brain axis, provide neuroprotective effects, and improve the long-term quality of life for patients. Therefore, such polypharmacy is like dopamine replacement therapy combined with FMT may reduce the content of α-Synuclein in the brain of PD patients to improve the motor and non-motor symptoms of PD patients. This article summarizes the latest mechanism by which gut microbiota participate in the gut-brain axis of PD and reviews the relevant research progress of the use of FMT as an adjuvant treatment for PD.

## 2 Gut microbiota and the gut-brain axis

The human intestinal tract contains about 1,000 types of symbiotic microorganisms, including bacteria, fungi, and viruses, with bacteria being the majority and accounting for more than 90% (Lucidi et al., 2021). The gut microbiome encodes over 3 million genes, which is 100 times the number of human genes, often referred to as the “second human genome” (Grice and Segre, 2012). Among the gut microbiota, bacteria are the most abundant and widely studied organisms. The gut microbiota has a complex structure and is composed of four bacterial phyla: Firmicutes, Bacteroidetes, Actinobacteria, and Proteobacteria, listed in order of prevalence (Qin et al., 2010). Symbiotic relationships exist between these bacteria and their hosts, supporting physiological homeostasis. Healthy gut microbiota are essential for nutrient absorption (Wittwer et al., 2023), material metabolism (Lin and Medeiros, 2023), body development (Dominguez-Bello et al., 2019), immune enhancement (Donald and Finlay, 2023) and anti-aging (Yu L. et al., 2023).

**FIGURE 1:**
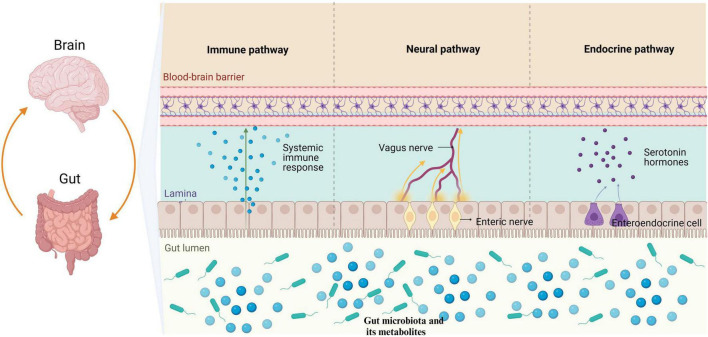
The gut microbiota has the ability to modulate the brain through three primary pathways: neural, endocrine, and immune. Specifically, the gut microbiota influences the enteric nervous system by transmitting information to the central nervous system via the vagus nerve. Furthermore, metabolites produced by gut microbiota have been found to stimulate enteroendocrine cells, leading to the release of hormones that play a regulatory role in brain function. Additionally, it is worth noting that the gut microbiota is capable of eliciting an immune response in the intestines, which subsequently results in the release of immune regulatory factors impacting physiological activities through mechanisms such as the BBB. BBB, blood-brain barrier.

As shown in Figure 1, the microbiota-gut-brain axis refers to a bidirectional communication pathway between the gut microbiota, the enteric nervous system, and the central nervous system. There are at least three parallel pathways through which the gut microbiota interacts with the brain: neural, endocrine, and immune (Agirman and Hsiao, 2021). The gut microbiota can transmit corresponding signals to brain regions directly via enteric nervous system, which innervate the vagus nerve and spinal cord afferent nerves, affecting physiological activities. The vagus nerve and spinal cord efferently project to the intestinal mucosa, directly affecting intestinal function and the composition of the gut microbiota (Agirman and Hsiao, 2021; Niesler et al., 2021). Metabolites of the gut microbiota and some subgroups can affect intestinal endocrine cells and induce the secretion of hormones and neurotransmitters that regulate the brain (Mulak, 2020; Agirman and Hsiao, 2021). Moreover, the gut microbiota interacts with neuroendocrine signaling pathways mediated by the hypothalamic-pituitary-adrenal (HPA) axis (Mulak, 2020).

Short-chain fatty acids (SCFAs) and lipopolysaccharides produced by the gut microbiota are some of the metabolites that can shape intestinal immune homeostasis. Gut microbiota interactions with local immune cells can lead to functional changes and promote peripheral immune effector cells to enter systemic circulation through the blood-brain barrier (BBB), causing neuroinflammation in the brain (Cheng et al., 2023a). In addition to the bottom-up regulation of brain pathological changes by the gut microbiota, some diseases of the central nervous system can also result in alterations to the gut microbiota, further aggravating conditions such as stroke (Peh et al., 2022). The communication mechanisms of the microbiota-gut-brain axis are complex. In addition, the relevant literature also suggests that the gut microbiota can be a key role in the pathogenesis of neurodegeneration by affecting neuromocytomosis, folding and removing protein errors, and the integrity of BBB (Padhi et al., 2022).

## 3 The specific mechanism of gut microbiota participation in the gut-brain axis of PD

### 3.1 The association between gut microbiota and α-synuclein

The abnormal aggregation of α-synuclein in the substantia nigra is a crucial pathological feature of PD, and understanding the relationship between gut microbiota and α-synuclein is key to clarifying the pathogenesis of PD. In the early stages of PD, gastrointestinal dysfunctions such as constipation, dysphagia, salivation, delayed gastric emptying, and anorectal dysfunction occur, often preceding motor symptoms (Travagli et al., 2020; Talman and Safarpour, 2023). Researchers have proposed that intestinal lesions may precede brain lesion, introducing the concept of body priority (Nuzum et al., 2022). In fact, α-synuclein is not only found in the brain but also present in the peripheral nervous system. In Shannon et al. (2012a,b) first identified abnormal aggregation of α-synuclein in the colonic submucosal nerve fibers of patients with PD. Subsequent research confirmed, using different PD models, that abnormal aggregation of α-synuclein in Schwann cells in the intestine leads to gastrointestinal dysfunction and vagus inflammation via the toll-like receptor-2 (TLR2)/myeloid differentiation primary response gene 88 (MyD88)/nuclear factor kappa-B (NF-κB) signaling pathway (Cheng et al., 2023c; Jiang et al., 2023). The current hypothesis is that α-synuclein is transmitted to the central nervous system through the vagus nervous system in a prion-like manner (Jan et al., 2021).

Similar to prions, α-synuclein has the ability to escape from a cell and subsequently spread into neighboring cells. Both *in vivo* and *in vitro* experiments have confirmed that transplanted cells can detect host α-synuclein (Desplats et al., 2009; Hansen et al., 2011). For example, Holmqvist et al. (2014) administered various forms of recombinant α-synuclein as well as α-synuclein derived from human PD brain lysates directly into the intestinal wall of rats, detecting α-synuclein in the vagus nerve 48 h later, eventually reaching the dorsal motor nucleus of the vagus nerve in the brain stem. Another study by Kim et al. (2019) injected pathological α-synuclein prefabricated fibers into the duodenum and muscular layer of the pylorus, tracking the diffusion path of this pathological α-synuclein in the brain. It first appeared in the posterior cerebral tail, then in the basolateral amygdala, dorsal raphe nucleus, and finally in the substantia nigra (Kim et al., 2019). Related studies have shown that the inoculation of pathological α-Synuclein prefabricated fibers can directly affect the activation of microglia in the brain (Rey et al., 2016; Manfredsson et al., 2018; Uemura et al., 2019). These studies strongly suggest that enteric-derived α-synuclein can migrate to the brain through the vagus nerve. Abnormal aggregation of α-synuclein in the nigrostriatum can further lead to M1-like phenotype polarization of microglia, activation of nucleotide oligomerization domain (NOD)-like receptor heat protein domain protein 3, and impaired autophagy and phagocytosis of microglia (Wood, 2022; Lv et al., 2023).

α-synuclein can accumulate and migrate in the intestine, and intestinal microorganisms are also associated with its production. Amyloid fibrin (curli) is a major component of bacterial biofilms, enhancing bacterial adhesion, colonization, and invasion, thus increasing virulence. Curli is often produced by *Salmonella* spp and *Escherichia coli*. It is formed by seven subunits, with CsgA serving as the primary structural subunit, and α-synuclein structurally similar to curli (Sheng et al., 2020; Yan et al., 2020). In rodent models, transgenic mice overexpressing α-synuclein were colonized with curli-producing *Escherichia coli* via artificial gavage, resulting in significantly increased α-synuclein in the intestine and brain compared to controls (Sampson et al., 2020; Schmit et al., 2023). Wang C. et al. (2021) found through whole genome screening that the *Escherichia coli* gene CsgA is a crucial gene that exacerbates neuropathy in a PD model of C. elegans. However, the exact transition mechanism remains unclear, and whether other bacteria produce curli remains to be elucidated. Notably, targeted reduction of curli-producing bacteria could offer a breakthrough in developing new therapies.

### 3.2 Role of SCFAs

SCFAs are a type of saturated fatty acids that contain fewer than six carbon atoms, including formic acid, acetic acid, propionic acid, butyric acid, and isobutyric acid. They are metabolic byproducts of gut microbiota, generated via the fermentation of polysaccharides, such as dietary fiber and resistant starch (Mirzaei et al., 2021). Importantly, 95% of SCFAs are transported and absorbed by colon epithelial cells, with only a small portion reaching peripheral tissues through systemic circulation. These SFCAs act as signaling molecules to regulate physiological functions or provide energy to target cells (Dalile et al., 2019). As signaling molecules, SCFAs mainly bind to G-protein coupled receptors (GPCRs) on cell membranes to regulate the host’s metabolic immune response, cell proliferation, and other physiological activities (He et al., 2020). Studies have shown that the fecal matter of patients with PD contains a higher abundance of certain SCFAs-producing bacteria (such as Prevotellaceae, Fusicatenibacter, Faecalibacterium, and Blautia) and the concentration of SCFAs decrease, correlating with the significant severity of clinical symptoms (Tan et al., 2021; Nishiwaki et al., 2022; Yang et al., 2022). This suggests that SCFAs may potentially play a significant role in the pathogenesis of PD.

Excessive activation of microglia in the substantia nigra and basal ganglia of patients with PD induces increased expression of inflammatory factors and macrophage infiltration, linking microglia activation and neuroinflammation to the pathogenesis of PD (Kwon and Koh, 2020). SCFAs, particularly butyric acid, seem to play a significant role in regulating the activation of microglia. Multiple studies have demonstrated that butyric acid has the potential to decrease the activation of astrocytes and microglia in the brains of mice with PD, inhibit the expression of inflammatory factors, upregulate brain- and glia-derived neurotrophic factors, and reduce dopaminergic neuron loss (Srivastav et al., 2019; Hou Y. et al., 2021; Guo et al., 2023; Ji et al., 2023). Moreover, the neuroprotective effect of butyric acid on PD may be mediated through the Janus kinase-2/signal transducer and activator of tran-ions-3 (JAK2/STAT3) and TLR4/MyD88/NF-kB signaling pathways (Guo et al., 2023; Ji et al., 2023).

The permeability of the BBB also influences PD progression. Reduced permeability allows inflammatory mediators and toxins to enter, contributing to neuroinflammation and loss of dopaminergic neurons. Butyric acid supplementation has been shown to significantly improve the levels of the tight junction proteins occludin and zonula occludens-1 in *1-methyl-4-phenyl-1,2,3,6-tetrahydropyridine* (MPTP)-induced PD mouse models, maintaining the integrity of the BBB (Liu et al., 2017). SCFAs also play a direct role in regulating energy metabolism and insulin release through the stimulation of peptide YY (PYY) and glucagon-like peptide 1 (GLP-1) secretion in intestinal endocrine cells (Larraufie et al., 2018; May and den Hartigh, 2021). Sun et al. (2021) found that supplementation with *Clostridium butyricum* in MPTP-induced PD mice increased the number of SCFA-producing bacteria, promoted GLP-1 secretion by intestinal endocrine L cells, and activated the GLP-1 receptor in the brain, thereby improving dopaminergic neuron loss and neuroinflammation.

Additionally, studies have revealed a significant reduction in propionate levels in the feces of patients with PD. Supplementation with propionate in PD mice was found to improve motor symptoms, possibly by enhancing intestinal epithelial barrier function through the Automatischer Kassentresor (AKT) signaling pathway (Huang et al., 2021). Osteocalcin has been shown to increase Bacteroides levels in the gut microbiota of PD mice, boost fecal propionate levels, and alleviate motor deficits and the loss of dopaminergic neurons (Hou Y. F. et al., 2021). Therefore, the therapeutic effects of propionic acid in PD warrant further investigation.

Accumulated evidence suggests that SCFAs can benefit PD by alleviating BBB damage, inhibiting microglial activation, and improving neuroinflammation. However, conflicting results also exist: butyric acid has been found to promote the activation of microglia and astrocytes, leading to an upregulation of pro-inflammatory factors such as interleukin-6, interleukin-18, inducible nitric oxide synthase, and nitric oxide, thereby aggravating neuroinflammation (Qiao et al., 2020). These contrasting outcomes may result from differences in model types, treatment durations, dosages, and experimental protocols. Therefore, SCFAs have great potential as safe and effective targets for the treatment of PD.

### 3.3 Other metabolites produced by the gut microbiota

One of the pathological characteristics of PD is the degeneration of dopaminergic neurons, and the most important current treatment is oral levodopa, which is absorbed from the intestinal tract to the systemic circulation, crosses the BBB, and is transformed into dopamine within the brain for therapeutic purposes (Lewitt, 2008). However, previous studies have found that *Enterococcus faecalis* (*E. faecalis*) in the intestine is also involved in the absorption and metabolism of levodopa (van Kessel et al., 2019; Wang Y. et al., 2021). The metabolism of levodopa and dopamine in patients with PD is correlated with the abundance of *E. faecalis*. The bacteria produce tyrosine decarboxylase, which has the ability to transform levodopa into dopamine, thereby weakening the efficacy of levodopa (van Kessel et al., 2019). Conversely, other studies have found that oral administration of berberine in PD model mice can increase the enzymatic activity of tyrosine hydroxylase, which acts as the rate-limiting enzyme in *E. faecalis*, thereby promoting the production of levodopa and alleviating PD symptoms (Wang Y. et al., 2021). This suggests that *E. faecalis* reduces the bioavailable levodopa, thereby limiting the amount of drug that reaches the brain and provides therapeutic benefit. but its biological activity on levodopa still requires further study.

Molecular hydrogen (H_2_), a metabolite of the gut microbiota, is usually produced by *Clostridium*, anaerobic cocci, and Enterobacteriaceae (Smith et al., 2019). Recent studies have indicated that the quantity of H_2_-producing bacteria in the intestines of patients with PD is significantly reduced, with H_2_ content being 2.2 times lower than that in healthy controls (Suzuki et al., 2018). In fact, earlier studies found that drinking water with low concentrations of H_2_ can mitigate the loss of dopaminergic neurons caused by MPTP, thus ameliorating symptoms in a mouse model of PD (Fujita et al., 2009). The underlying mechanism may be related to the antioxidant, anti-inflammatory and neuroprotective properties of H_2_ (Ostojic, 2018).

Hydrogen sulfide (H_2_S) is a gaseous neurotransmitter, and excessive H_2_S produced by intestinal bacteria may induce PD. Sulfate-reducing bacteria are major producers of H_2_S in the feces of healthy individuals (Dordevic et al., 2021). Importantly, bacterial species known to produce H_2_S, such as Helicobacter pylori, *Clostridium difficile*, and *Vibrio desulfuricum*, are reportedly associated with the occurrence of PD (Huang et al., 2018; Kang et al., 2020; Murros et al., 2021; Nie et al., 2023). Excessive endogenous H_2_S induces the release of cytochrome C (Cyt c) into the mitochondria of intestinal cells and increases cytoplasmic iron levels, thereby increasing reactive oxygen species (ROS) (Murros, 2022). The presence of α-synuclein, Cyt c and ROS together leads to the aggregation of α-synuclein in the intestine and its transmission to the brain, promoting the development of PD (Murros, 2022). However, exogenous inhalation of H_2_S or injection of NaHS (an H_2_S donor) has been found to have a beneficial effects on PD in both cell and animal experiments (Kida et al., 2011; Sarukhani et al., 2018; Tian et al., 2022; Hacioglu et al., 2023; Nagashima et al., 2023), possibly due to the upregulation of antioxidant protein-coding genes (Kida et al., 2011). Therefore, the role of H_2_S in PD needs further study, as differences in H_2_S concentration, the method of administration methods, and the target sites may lead to varying results. Moreover, studies suggest that low concentrations of H_2_S inhalation are beneficial for stroke recovery, whereas high concentrations are beneficial for other conditions (Chan and Wong, 2017).

**FIGURE 2:**
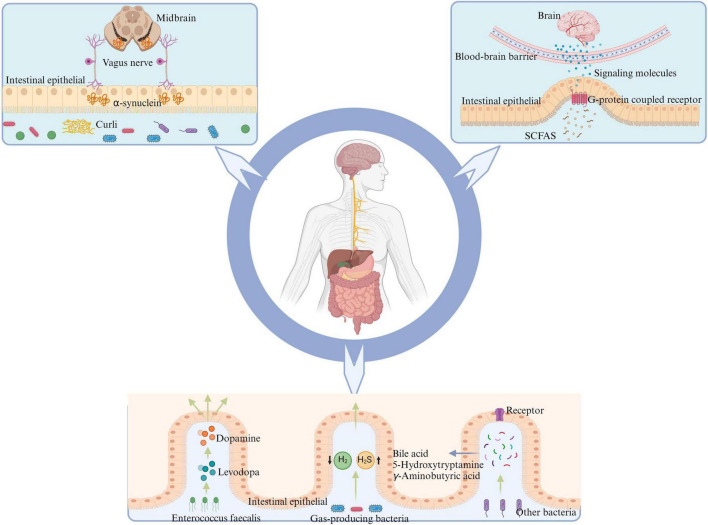
Gut microbiota has the potential to influence the onset of PD through various mechanisms: certain intestinal bacteria promote α-synuclein aggregation in the intestine by producing curli, leading to PD via transmission through the vagus nerve to the nigrostriatum of the midbrain. SCFAs, produced by intestinal flora metabolism, activate intestinal G-protein-coupled receptors, promoting intestinal cells to release corresponding signaling molecules and alleviate neuroinflammation crossing the BBB. *E. faecalis* in the intestine can impact the conversion between levodopa and dopamine, thereby affecting PD symptoms. Reduction in the population of H_2_-producing bacteria and an increase in H_2_S-producing bacteria within the intestine may lead to alterations in the levels of related gaseous molecules, thereby impacting PD through effects on both the intestinal barrier and BBB. Other metabolites of gut microbiota may further contribute to the advancement and progression of PD by interacting with corresponding receptors on intestinal cells, such as bile acids, 5-hydroxytryptamine, and gamma-aminobutyric acid. PD, Parkinson’s disease; SCFAs, short-chain fatty acids; BBB, blood-brain barrier; H_2_, molecular hydrogen; H_2_S, hydrogen sulfide.

In conclusion, as depicted in Figure 2, the gut microbiota and its metabolites are involved in the pathogenesis of PD. However, the roles of some important metabolites in PD need further clarification through additional studies. Furthermore, bacterial metabolites such as bile acids (Zangerolamo et al., 2021), 5-hydroxytryptamine (Frouni et al., 2022) and γ-aminobutyric acid (Roberts et al., 2021) are also involved in the pathological progression of PD.

## 4 Characteristics of gut microbiota in Parkinson’s disease

Numerous studies have demonstrated differences in gut microbiota between patients with PD and healthy controls across various classification levels. Table 1 summarizes the findings of 13 studies chronologically. Decreased relative abundances in PD include Prevotellaceae (Scheperjans et al., 2015; Unger et al., 2016; Bedarf et al., 2017; Mertsalmi et al., 2017; Petrov et al., 2017; Babacan Yildiz et al., 2023), Roseburia (Barichella et al., 2019; Boktor et al., 2023) and Faecalibacterium (Petrov et al., 2017; Boktor et al., 2023), while increased abundances include Akkermansia (Bedarf et al., 2017; Heintz-Buschart et al., 2018; Barichella et al., 2019; Baldini et al., 2020; Zhang K. et al., 2022; Boktor et al., 2023), Verrucomicrobiaceae (Heintz-Buschart et al., 2018; Barichella et al., 2019; Zhang K. et al., 2022; Babacan Yildiz et al., 2023), Bifidobacterium (Hill-Burns et al., 2017; Petrov et al., 2017; Boktor et al., 2023) and Lactobacillus (Petrov et al., 2017; Baldini et al., 2020; Babacan Yildiz et al., 2023). These observations align with most of meta-analysis results (Nishiwaki et al., 2020; Hirayama and Ohno, 2021; Toh et al., 2022; Zhou et al., 2023). A study by Scheperjans et al. (2015) first reported a 77.6% reduction in Prevotellaceae abundance in PD patients. However, the exact relationship between Prevotellaceae and PD remains unclear due to sequencing method limitations, although Prevotellaceae is known to generate beneficial H_2_S (Cakmak, 2015).

**TABLE 1:** Characteristics of gut microbiota and clinical significance of microbiome variation in PD.

Subgroups and sequencing methods	Decreased taxa	Increased taxa	Clinical significance of microbiome variation	Country	References
PD: *N* = 72 HC: *N* = 72 16S rRNA (V1-V3)	Prevotellaceae		The prevalence of Enterobacteriaceae demonstrated a positive correlation with the severity of postural instability and gait difficulty.	Finland	Scheperjans et al., 2015
PD: *N* = 34 HC: *N* = 34 qPCR	Bacteroidetes, Prevotellaceae	Enterobacteriaceae		Germany	Unger et al., 2016
PD: *N* = 89 HC: *N* = 66 16S rRNA (V3-V4)	Dorea, Bacteroides, Prevotella, Faecalibacterium, Stoquefichus massiliensis, Blautia glucerasea, Coprococcus eutactus, and Ruminococcus callidus	Christensenella, Catabacter, Lactobacillus, Oscillospira, Bifidobacterium, Ruminococcus bromii, and Papillibacter cinnamivorans		Russia	Petrov et al., 2017
PD: *N* = 197 HC: *N* = 130 16S rRNA	Lachnospiraceae, Pasteurellaceae, Verrucomicrobiaceae	Bifidobacteriaceae, Lactobacillaceae, Tissierellaceae, Christensenellaceae	Microbiome variation may be related to the metabolism of botanical compounds and the degradation of xenobiotics.	USA	Hill-Burns et al., 2017
PD: *N* = 74 HC: *N* = 75 16S rRNA (V1-V3)	Prevotella		The lower abundance of Prevotella bacteria in PD patients with irritable bowel syndrome.	Finland	Mertsalmi et al., 2017
PD: *N* = 31 HC: *N* = 28 Shotgun metagenomics	Prevotella copri, *Clostridium saccharolyticum*, Eubacterium biforme	Alistipes shahii, Akkermansia muciniphila		Germany	Bedarf et al., 2017
PD: *N* = 76 iRBD: *N* = 21 HC: *N* = 78 16S and 18S rRNA (V4)	Melainabacteria	Verrucomicrobia, Verrucomicrobiales, Verrucomicrobiaceae, Akkermansia, Prevotella	Eighty percent of the distinct gut microbes in PD compared with iRBD were identified. In PD: Motor symptoms related to Anaerotruncus spp., *Clostridium* XIVa, and Lachnospiraceae, Non-motor symptoms related to Anaerotruncus, Akkermansia, and several unclassified Bacteria.	Germany	Heintz-Buschart et al., 2018
PD: *N* = 45 HC: *N* = 45 16S rRNA (V3-V4)	Lactobacillus, Sediminibacterium	Genera *Clostridium* IV, Sphingomonas, Aquabacterium, *Clostridium* XVIII, Holdemania, Anaerotruncus, Butyricicoccus	The presence of *Escherichia*/*Shigella* showed a negative correlation with the duration of disease, while Dorea and Phascolarctobacterium demonstrated a negative association with levodopa equivalent doses. Additionally, Butyricicoccus and *Clostridium* XlVb were found to be linked with cognitive impairment.	China	Qian et al., 2018
PD: *N* = 193 HC: *N* = 113 16S rRNA (V3-V4)	Roseburia, Lachnospiraceae, Ruminococcus	Enterobacteriaceae, Akkermansia, Verrucomicrobiaceae, Lactobacillaceae, Oscillospira	Lachnospiraceae (including Roseburia) exhibited a negative association with disease duration, while Lactobacillaceae (including Lactobacillus) and Akkermansia (as well as Verrucomicrobia and Verrucomicrobiaceae) showed a positive association with disease duration. Patients with higher abundance in Christensenellaceae demonstrated more severe non-motor symptoms.	Italy	Barichella et al., 2019
PD: *N* = 147 HC: *N* = 162 16S rRNA (V3-V4)	Turicibacter	Anaerotruncus, Christensenella, Lactobacillus, Streptococcus, Akkermansia, Bilophila	The Hoehn and Yahr staging revealed a negative association with Paraprevotella, while showing a positive association with Bilophila.	Luxembourg	Baldini et al., 2020
PD: *N* = 96 HC: *N* = 74 16S rRNA (V4)		Proteobacteria, Actinobacteria, Enterococcus, Verrucomicrobiota, Akkermansia, Ruminococcaceae, Hungatella	Extended duration of PD was found to be correlated with decreased levels of the Synergistota phylum and significant shifts in six genera. There were increased levels of Fournierella, DTU089, and Haemophilus, as well as decreased levels of Pseudomonas, Lactobacillus, and Roseburia. In addition, two genera were linked to higher UPDRS III scores: an increased level of Lachnospiraceae_NK4B4_ group and a decreased level of Senegalimassilia.	USA	Zhang K. et al., 2022
Two independent cohorts PD, *N*1 = 48, *N*2 = 47, household controls, *N*1 = 29, *N*2 = 30, healthy population controls, *N*1 = 41, *N*2 = 49 Shotgun metagenomics	Faecalibacterium, Roseburia genera, Faecalibacterium prausnitzii, Eubacterium	Actinobacteria, Bifidobacterium bifidum, Eisenbergiella tayi, Ruthenibacterium lactatiformans, Akkermansia muciniphila		USA	Boktor et al., 2023
PD: *N* = 42 HC: *N* = 42 16S rRNA (V3-V4)	Firmicutes, Coriobacteriales Incertae Sedis, Lachnospiraceae ND3007 group, Eubacterium hallii group, Tyzzerella, Ruminococcus gauvreauii group, Fusicatenibacter, Prevotella	Verrucomicrobiota Lactobacillaceae, Akkermansiaceae, Lactobacillus		Turkey	Babacan Yildiz et al., 2023

PD, Parkinson’s disease; HC, healthy control; 16S rRNA, 16S ribosomal RNA; qPCR, quantitative real-time PCR; iRBD, isolated REM sleep behavior disorder; UPDRS, Unified Parkinson’s Disease Rating Scale.

Bacteria that increase in PD, such as Akkermansia, Bifidobacterium, and Lactobacillus, are often considered probiotics (Cani, 2018), with their increased abundance seen as an adaptive response to PD (Tan et al., 2022). Moreover, many studies link gut microbiota with disease severity, duration, drug treatment, motor symptoms and non-motor symptoms to explain disease development (Bi et al., 2022). Associations include Enterobacteriaceae abundance positively correlating with postural instability and gait difficulty severity (Scheperjans et al., 2015), while *Escherichia*/*Shigella* were negatively associated with disease duration (Qian et al., 2018), and Dorea and Phascolarctobacterium negatively associated with levodopa equivalent doses (Qian et al., 2018). Paraprevotella has shown a negative association, while Bilophila demonstrated a positive correlation with Hoehn and Yahr staging (Baldini et al., 2020). However, there was a notable disparity in the composition of gut microbiota between the PD and healthy individuals. Although significant differences in gut microbiota composition exist between PD and control groups, heterogeneity among studies, including feces collection processes, control selection, sequencing methods, geographical environments, diets, and medications complicates identifying unified bacterial biomarkers or constructing early diagnostic models. Recently, researchers proposed a potential diagnostic biomarker for PD based on a shotgun metagenomic sequencing-derived PD index, unaffected by disease severity or PD medication use (Qian et al., 2020). This index holds promise as a new diagnostic tool for distinguishing PD.

While single omics technologies can reflect species and genera differences in gut microbiota, multiple omics combinations are needed to understand specific pathogenic mechanisms. Therefore, researchers often integrate gut microbiome with fecal metabolomics, transcriptomics, and proteomics to explore the intricate role of the microbiota in human health. For instance, Tan et al. (2021) correlated 16S rRNA gene sequencing with fecal metabolomics, revealing decreased bacteria producing SCFAs and significant fecal butyrate reduction in PD. Cirstea et al. (2020) investigated gut microbiome functions via gut microbiome and serum metabolomics correlation, and observed a reduction in carbohydrate fermentation capacity and butyric acid synthesis, as well as an increase in proteolytic fermentation and the production of harmful amino acid metabolites in PD. Despite these insights, most current studies are cross-sectional and cannot definitively establish causal relationships between the gut microbiome and PD. Longitudinal studies using multi-omics approaches are thus warranted for deeper understanding. During a two-year longitudinal follow-up study, Aho et al. (2019) found that the gut microbiota of both patients with PD and healthy controls exhibited no significant changes across various time points, suggesting the need for longer follow-ups for comprehensive insights. In conclusion, prospective or longitudinal studies employing multi-omics approaches are essential for unraveling the gut microbiota-PD relationship.

## 5 Application of fecal microbiota transplantation in Parkinson’s disease

FMT involves the transfer of gut microbiota from a healthy donor into the intestines of a patient, thereby reshaping the recipient’s gut microbiota to a normalized state. This novel therapy has emerged in recent years as a means to modulate the gut microbiota. Because of its notable efficacy and safety, FMT holds promise not only in treating intestinal disorders such as *Clostridium difficile* infection (Walter and Shanahan, 2023), inflammatory bowel disease (Haifer et al., 2022), and irritable bowel syndrome (Holvoet et al., 2021), but also in exploring novel avenues for managing systemic conditions including non-alcoholic fatty liver disease (Gupta et al., 2021), obesity (Rinott et al., 2021), type 2 diabetes (Wu et al., 2022), autoimmune pancreatitis (Kamata et al., 2019), and neoteric diseases (O’Leary, 2021).

The gut microbiota exerts influence on brain function through the gut-brain axis, and FMT has demonstrated efficacy in treating certain central nervous system disorders (Matheson and Holsinger, 2023). Therefore, this review aims to provide an overview of the current use of FMT in animal models of PD and human studies, and is summarized in Tables 2, 3, respectively, to provide insights and conclusions about the potential of FMT as a therapeutic intervention for PD.

**TABLE 2:** FMT in animal studies on PD.

Model	Experimental design	Outcomes	References
12–13 weeks, Germ-free Thy1-αSyn mice, *N* = 3–6 per group	Donors: PD patients or healthy controls FMT: single infusion via oral gavage	Increased motor impairment was observed following FMT, with minimal changes in weight and gastrointestinal function.	Sampson et al., 2016
8-week-old, MPTP-treated C57BL/6 mice, *N* = 15 per group	Donors: control mice FMT: oral gavage once daily for 7 days.	Reshaped gut microbiota, decreased fecal SCFAs, alleviated physical impairment, reduced the activation of microglia and astrocytes in the substantia nigra, increased striatal dopamine and 5-hydroxytryptamine, suppressed TLR4/TNF-α signaling pathway.	Sun et al., 2018
8-week-old, MPTP-treated C57BL/6 mice, *N* = 10 per group	Donors: control mice FMT: oral gavage once daily for 7 days.	Alleviated physical impairment, decreased fecal SCFAs and expression of α-synuclein, inhibited the activation of microglia, suppressed TLR4/TNF-α signaling pathway.	Zhong et al., 2021
8-week-old, Rotenone-treated C57BL/6 mice, *N* = 15 per group	Donors: control mice FMT: oral gavage once daily for 14 days.	Restored the gut microbiota dysbiosis, decreased the gastrointestinal dysfunctions and inflammation, Alleviated gastrointestinal barrier destruction and BBB impairment, ameliorated neuroinflammation and the motor deficits, suppressed TLR4/TNF-α signaling pathway.	Zhao et al., 2021
6–8 weeks, MPTP + probenecid-treated C57BL/6 mice, *N* = 6 per group	Donors: control mice FMT: oral gavage once daily for 14 days.	Restored the gut microbiota dysbiosis, reduced level of inflammation in the substantia nigra, alleviated motor dysfunction, reduced the activation of microglia and astrocytes.	Zhang T. et al., 2022
10-week-old, MPTP-treated C57BL/6 mice, *N* = 12 per group	Donors: PD patients or healthy controls FMT: oral gavage once daily for 10 days.	Fecal microbiota from healthy controls improved gut microbiota dysbiosis, neurodegeneration, microgliosis, astrogliosis, mitochondrial impairments through the AMPK/SOD2 pathway, degeneration of nigrostriatal pericytes, and BBB integrity. fecal microbiota from PD patients revealed the opposite result	Xie et al., 2023
Adult male 6-OHDA-treated Wistar rats, *N* = 8 per group	Donors: control rats FMT: oral gavage once daily for 14 days.	Increased NMNAT2 expression, relieved neurobehavioral deficits, reduced oxidative stress including: GSH content, GSHPx activity and SOD activity.	Yu J. et al., 2023
8-week-old, MPTP-treated C57BL/6 mice, *N* = 17 per group	Donors: young (7 weeks) and aged (23 months) male C57BL/6 J mice FMT: oral gavage once daily for 7 days.	Fecal microbiota from aged mice improved including locomotor function, the level of neurotransmitters in the striatum, the loss of dopaminergic neurons, gut microbiota dysbiosis, fecal SCFAs levels and neurogenesis in the hippocampus.	Qiao et al., 2023

PD, Parkinson’s disease; FMT, fecal microbiota transplantation; SCFAs, short-chain fatty acids; TLR4, toll-like receptor-4; TNF–α, tumor necrosis factor-alpha; BBB, blood-brain barrier; AMPK, AMP-activated protein kinase; NMNAT2, nicotinamide mononucleotide adenylyltransferase 2; GSH, glutathione; SOD, superoxide dismutase; GSH-Px, glutathione peroxidase; MPTP, 1-methyl-4-phenyl-1,2,3,6-tetrahydropyridine.

**TABLE 3:** FMT in human studies on PD.

Patient and donor	Type of clinical study	Therapeutic schedule	FMT-effect	Side-effects	Reference
Patient: a 71-year-old male with PD Donor: a 26-year-old healthy man	case report	200 mL of fecal microbiota suspension, Once daily for a period of 3 days, Transendoscopic enteral tube, Three-month follow-up	The tremor nearly disappeared one week after FMT but returned in the right lower extremity two months later. Constipation significantly improved until the end of the follow-up.	NO side-effects	Huang et al., 2019
Patient: 15 PD patients (49 to 72 years old) Donor: healthy human (18 to 24 years old)	Pilot Study	Single infusion, 5 patients delivered via nasoduodenal tube, 10 patients delivered via colonoscopy, One-year follow-up	Decreased the score of PSQI, HAMD, PDQ-39, NMSQ, HAMA, UPDRS III at 1 and 3 months after FMT, FMT via colonoscopy was more effective than nasointestinal tube.	Diarrhea, abdominal pain and flatulence	Xue et al., 2020
Patient: 11 PD patients (40 to 84 years old) Donor: China fmtBank	Evaluation Research	Nasoduodenal tube, Twelve-week follow-up	NMSS, PAC-QOL, UPDRS, and Wexner constipation scores increased.	Mild diarrhea, abdominal pain, venting, flatulence, nausea, and throat irritation	Kuai et al., 2021
Patient: 6 PD patients (47 to 73 years old) Donor: a 38-year-old man and a 50-year-old man.	Case Series	300 mL of fecal microbiota suspension delivered via colonoscopy, Twenty-four-week follow-up	NMSS, UPDRS III and Wexner constipation scores increased.	Recurrent episodes of vasovagal pre-syncope	Segal et al., 2021
Patient: 27 PD patients (30 to 85 years old) Donor: 4 stool donors	Double-blind Randomized Trial	Sixteen capsules of FMT by oral administration, once a week for 3 consecutive weeks, twelve-week follow-up	MDS-UPDRS total scores increased, improved gastrointestinal disorders	Nausea, flatulence, diarrhea	Cheng et al., 2023b

PD, Parkinson’s disease; FMT, fecal microbiota transplantation; PSQI, Pittsburgh sleep; HAMD, Hamilton Depression Rating Scale; HAMA, Hamilton Anxiety Rating Scale; PDQ-39, 39-item Parkinson’s Disease Questionnaire; NMSQ, Non-Motor Symptoms Questionnaire; UPDRS, Unified Parkinson’s Disease Rating Scale; NMSS, Non-Motor Symptoms Scale; PAC-QOL, Patient Assessment of Constipation-Quality of Life; MDS-UPDRS, Movement Disorder Society Unified-Parkinson’s Disease Rating Scale.

### 5.1 Animal studies on Parkinson’s disease

Sampson et al. (2016) initially established a Germ-free Thy1-αSyn mouse model, and subsequently introduced fecal samples from patients with PD versus healthy controls via gavage into these germ-free mice. Their findings revealed that the Thy1-αSyn mice receiving fecal transplants from patients with PD exhibited more pronounced motor function impairment compared to the group receiving fecal transplants from healthy controls. However, minimal changes were observed in body weight or gastrointestinal function (Sampson et al., 2016). Additionally, this study highlighted that SCFAs exacerbated α-synuclein aggregation in the brain and motor deficits in α-synuclein-overexpressing mice (Sampson et al., 2016). Notably, this study pioneered the use of FMT to explore the interplay between gut microbiota, its metabolites, and PD in a germ-free mouse model. Subsequent research further corroborated the direct therapeutic potential of FMT in PD mouse models (Sun et al., 2018; Zhao et al., 2021; Zhong et al., 2021; Zhang T. et al., 2022).

Sun et al. (2018) demonstrated that FMT could modulate the gut microbiota of MPTP-induced PD mice, leading to reduced SCFAs levels in feces, which in turn improved motor function while decreasing microglia and astrocytic activity in the substantia nigra. Moreover, FMT was found to decrease α-synuclein levels in the brains of PD mouse models (Zhong et al., 2021) and enhance gut and BBB permeability (Zhao et al., 2021). Mechanistic investigations into FMT therapy suggested that these effects may occur through inhibition of the TLR4/tumor necrosis factor-alpha (TNF-α) signaling pathway (Sun et al., 2018; Zhao et al., 2021; Zhong et al., 2021). Further clarification of the role of TLR4 in PD was provided by Perez-Pardo et al. (2019), where TLR4 knockout in PD mice reduced intestinal and brain inflammation, implicating TLR4 as a key receptor in PD-related neurodegeneration.

In these studies, donors were healthy mice of the same strain. To explore the therapeutic potential of human-derived gut microbiota in PD models, Xie et al. (2023) transplanted fecal samples from patients with PD and healthy individuals into MPTP-induced mice. Their results indicate that FMT from healthy human donors suppressed microgliosis and astrogliosis, restored nigra peristriatal cells, and preserved BBB integrity, possibly mediated via the AMP-activated protein kinase (AMPK)/superoxide dismutase 2 (SOD2) signaling pathway to mitigate mitochondrial damage (Xie et al., 2023). Yu J. et al. (2023) utilized bioinformatics analysis to identify the significance of nicotinamide mononucleotide adenylyltransferase 2 (NMNAT2) in PD pathogenesis induced by gut microbiota. Further investigation into the molecular mechanisms of FMT in PD treatment involved treating 6-OHDA-treated rats with FMT, resulting in upregulation of NMNAT2 expression. This upregulation, along with reduced glutathione (GSH) content, total SOD, and glutathione peroxidase (GSH-Px) activities, contributed to the mitigation of oxidative stress and amelioration of neurobehavioral defects in rats (Yu J. et al., 2023).

Regarding donor selection, most studies utilized fecal samples from young and healthy individuals, with minimal investigation into the fecal samples from elderly donors for FMT. Qiao et al. (2023) observed that while fecal transplantation from old and young mice did not alter the intestinal tract or neuroinflammation in PD mice, fecal samples from old mice improved motor disorders by enhancing neurogenesis in the hippocampus. Similar effects on the hippocampus of elderly mice were reported in prior studies (Rei et al., 2022), underscoring the importance of considering donor when performing FMT on PD patients.

In summary, these findings indicate that FMT holds promise in treating PD by mitigating inflammation via TLR4/TNF-α signaling pathway inhibition, improving mitochondrial damage through AMPK/SOD2 pathway activation, and reducing oxidative stress via NMNAT2 upregulation. Future research endeavors should further elucidate the therapeutic mechanisms of FMT in PD.

### 5.2 Human studies on Parkinson’s disease

Although FMT has not been widely implemented in treating PD, several case reports and case-control studies suggest that FMT can normalize gut microbiota disturbances in patients with PD, leading to improvements in both motor and non-motor symptoms (Huang et al., 2019; Xue et al., 2020; Kuai et al., 2021; Segal et al., 2021). In Huang et al. (2019) conducted a pioneering study applying FMT to a patient with PD suffering from constipation. They inserted a transendoscopic enteral tube into the patient’s ileocecal area via colonoscopy, administering 200 ml of fecal bacterial solution daily for three days. During a three-month follow-up, the patient’s bowel movement time decreased from > 30 min to < 5 min. Remarkably, the patient’s resting tremor in both lower limbs nearly disappeared one week post-treatment, although symptoms in the right lower limb reappeared two months later without any adverse reactions (Huang et al., 2019). While this was merely a case report, it highlighted the potential of FMT in the management of PD.

In Xue et al. (2020) expanded on this research by applying FMT to a larger cohort of patients with PD. They conducted a self-controlled clinical study involving ten patients with PD who underwent FMT via colonoscopy and five patients with PD who underwent FMT via a nasojejunal tube. The results indicated improvements in motor function, anxiety, depression, and sleep symptoms among patients treated via colonoscopy, whereas those treated via the nasojejunal tube exhibited less significant improvements (Xue et al., 2020). This suggests that the route of FMT administration may impact its therapeutic efficacy, a hypothesis further supported by Gundacker et al. (2017), who proposed that bacterial loss during migration from the jejunum to the colon could explain the variance in effectiveness.

Cheng et al. (2023b) conducted an FMT trial involving 56 patients with mild-to-moderate PD, administering fecal bacterial capsules. Throughout the duration of the follow-up period, patients receiving FMT exhibited significant improvements in autonomic symptoms and gastrointestinal disorders compared to those in the placebo group, without experiencing any serious adverse reactions. This study represents the only clinical randomized controlled trial reported to date (Cheng et al., 2023b). Collectively, these studies underscore the potential of FMT for the treatment of PD.

Safety is a paramount consideration in FMT applications. Reported side effects include diarrhea, abdominal pain, venting, flatulence, nausea, and throat irritation, which are usually mild and transient (Xue et al., 2020; Kuai et al., 2021). However, one patient in Segal’s study experienced recurrent episodes of vasovagal presyncope 24 h post-FMT, lasting for 8 h—an adverse effect not previously documented in FMT treatments (Segal et al., 2021). The longest follow-up duration in current studies is one year, which is insufficient to conclusively determine the long-term therapeutic effectiveness of FMT in PD treatment (Xue et al., 2020).

In summary, FMT has demonstrated therapeutic potential for PD, but more extensive and rigorous prospective studies are necessary to confirm its efficacy, safety and sustainability.

### 5.3 Deep thinking on the treatment of PD by FMT

Despite the promising results from animal and human studies on the treatment of PD with FMT, several key issues warrant deeper consideration, as they may significantly influence the efficacy of FMT.

First, the source of fecal donors is crucial for the treatment’s success. Donors can be either allogeneic or autologous. Allogeneic feces typically come from fecal banks or universal donors and undergo rigorous screening to ensure safety (Sorbara and Pamer, 2022). However, some pathogens may evade detection, posing potential risks post-transplantation. Therefore, autologous feces, stored before the onset of disease, might be a more suitable donor source (Sorbara and Pamer, 2022). Moreover, the age of allogeneic fecal donors is an important factor. Most current donors are healthy young adults, whose future risk of developing PD is unknown, which could inadvertently affect the progression of PD in recipients. Animal studies suggest that feces from elderly mice can enhance hippocampal neurogenesis and improve PD symptoms (Qiao et al., 2023), indicating that feces from healthy elderly donors might be more beneficial for FMT treatment.

The method of FMT administration is another critical consideration. Common methods include nasal jejunal tube transplantation, oral microflora capsule transplantation, and colonoscopy. Clinical studies have reported better outcomes with colonoscopic transplantation compared to nasojejunal tube transplantation (Xue et al., 2020). However, since PD patients are generally older and some may not tolerate colonoscopy, the balance between efficacy and safety is essential. Some studies also recommend using antibiotics like vancomycin to deplete the patient’s native gut microbiota, facilitating the colonization of donor microbiota (Contarino et al., 2023). However, the necessity of pre-antibiotic treatment remains undetermined, though animal experiments indicate that ceftriaxone might offer neuroprotective benefits in PD models (Zhou et al., 2021). More case-control studies are needed to evaluate the impact of antibiotic pretreatment on the efficacy of FMT.

Furthermore, there is no standardized FMT regimen for PD treatment. Variables such as the number of transplants and the interval between treatment cycles significantly influence outcomes. Most current cases report single infusions of bacterial fluid (Xue et al., 2020; Kuai et al., 2021; Segal et al., 2021), with one study administering daily infusions for three consecutive days (Huang et al., 2019). Symptom improvement, particularly for constipation, is more pronounced than motor symptom relief. Additionally, the longest follow-up duration in existing studies is one year (Xue et al., 2020), providing limited data on the long-term efficacy of FMT in PD treatment. Therefore, establishing a standard FMT regimen and determining the necessity of maintenance therapy are crucial areas for future research.

The ideal composition of the gut microbiota for PD treatment is still unclear. However, effective microbiota should possess anti-inflammatory properties, produce SCFAs, H_2_, and H_2_S, and should not interfere with anti-PD drug metabolism. Future developments in FMT may involve assembling well-characterized, functional bacterial strains into symbiotic microbiota to reestablish a healthy gut environment while avoiding potential pathogens (Sorbara and Pamer, 2022).

## 6 Conclusion and perspectives

In conclusion, the gut microbiota establishes a close interaction with PD through the microbiota-gut-brain axis. Disturbances in gut microbiota promote the development of PD by producing curli protein, reducing SCFAs production, and regulating other key bacterial metabolites. These changes contribute to the accumulation of α-synuclein in the intestine, its migration to the brain, decreased permeability of the intestinal and blood-brain barriers, and activation of microglia. This cascade ultimately leads to the abnormal aggregation of α-synuclein in the brains of patients with PD and the degeneration and necrosis of dopaminergic neurons in the substantia nigra.

There is a potential to use the gut microbial characteristics of patients with PD as biomarkers to assist in early diagnosis. FMT can rebuild the gut microbiota and play a neuroprotective role through anti-inflammatory mechanisms, improving mitochondrial function, alleviating oxidative stress, and effectively improving non-motor symptoms and some motor symptoms in patients with PD.

However, the focus should be on both the molecular mechanisms of FMT in treating PD and on conducting multicenter clinical studies to clarify its clinical effects. Currently, the number of relevant clinical studies is limited, and our understanding of the safety, effectiveness, and sustainability of FMT in treating PD is still evolving. Possible adverse reactions and long-term risks of FMT require careful attention, and the protocols for managing adverse effects should be prepared in advance.

To enhance the therapeutic effect of FMT, future research should aim to standardize key factors such as donor source, transplantation route, pre-transplantation antibiotic treatment, and standard transplantation protocols. It is crucial to examine research results critically and avoid overstating their significance. Rigorous and comprehensive studies are necessary to establish the causal relationship between gut microbiota and PD.

In conclusion, FMT demonstrates significant potential in the management of PD. More comprehensive and methodologically rigorous experiments are required to validate the effectiveness and safety of the treatment, ensuring that future applications are both effective and reliable.
